# The Neuronal Glutamate Transporter EAAT3 in Obsessive-Compulsive Disorder

**DOI:** 10.3389/fphar.2019.01362

**Published:** 2019-11-15

**Authors:** Angélica P. Escobar, Jens R. Wendland, Andrés E. Chávez, Pablo R. Moya

**Affiliations:** ^1^Centro Interdisciplinario de Neurociencia de Valparaíso CINV, Facultad de Ciencias, Universidad de Valparaíso, Valparaíso, Chile; ^2^Instituto de Fisiología, Facultad de Ciencias, Universidad de Valparaíso, Valparaiso, Chile

**Keywords:** EAAT3, OCD, obsessive-compulsive disorder, glutamate transporter, synaptic function, NMDAR, animal model, SLC1A1

## Abstract

Obsessive compulsive disorder (OCD) is a heterogeneous psychiatric disorder affecting 1%–3% of the population worldwide. About half of OCD afflicted individuals do not respond to currently available pharmacotherapy, which is mainly based on serotonin reuptake inhibition. Therefore, there is a critical need to search novel and improved therapeutic targets to treat this devastating disorder. In recent years, accumulating evidence has supported the glutamatergic hypothesis of OCD, and particularly pointing a potential role for the neuronal glutamate transporter EAAT3. This mini-review summarizes recent findings regarding the neurobiological basis of OCD, with an emphasis on the glutamatergic neurotransmission and EAAT3 as a key player in OCD etiology.

## Obsessive Compulsive Disorder

Obsessive compulsive disorder (OCD) is a psychiatric disorder affecting 1%–3% of the population worldwide (Grabe et al., 2000; Angst et al., 2004). It is characterized by recurrent, intrusive worries, feelings or unwanted thoughts (obsessions), and repetitive, structured, ritualistic mental acts and/or behaviors (compulsions). OCD is clinically heterogeneous, displaying a wide range of symptomatic expression (Murphy et al., 2013). Most OCD patients also have high levels of anxiety, likely due to the inability to control or stop the appearance of obsessions (Pauls et al., 2014). Both obsessions and compulsions are time consuming, causing a high impairment in social and occupational areas (American Psychiatric Association, 2013).

Current OCD treatment involves cognitive-behavioral therapy alone or combined with pharmacotherapy mainly based on serotonin reuptake inhibitors (SRI), such as fluoxetine, clomipramine or paroxetine (Bouvard et al., 2004; Cottraux et al., 2005; Skapinakis et al., 2016). However, only about half of patients achieve an adequate decrease in the severity of symptoms (Hirschtritt et al., 2017), highlighting the need for new therapeutic options to treat this devastating disorder.

## Cortico-Striato-Thalamo-Cortical Loop Modifications in OCD

Since original reports, neuroimaging studies have consistently indicated that a dysfunction in the cortico-striato-thalamo-cortical (CSTC) loop is involved in OCD (Milad and Rauch, 2012; Nakao et al., 2014; Burguiere et al., 2015). In a brief and oversimplified manner, the CSTC loop is composed of glutamatergic pyramidal cortical neurons that project onto striatal subnuclei. From here, GABAergic neurons project to basal ganglia and the thalamus throughout both direct and indirect pathways; in turn, the thalamus sends recurrent projections back to cortical areas (Alexander and Crutcher, 1990). A normal CSTC loop function is required for regulating habitual and goal-directed behaviors (Gerfen and Surmeier, 2011). Neuroimaging studies, for example, indicated changes in the volume of the anterior cingulate cortex and the thalamus in OCD patients compared to healthy controls (Atmaca et al., 2007; Radua and Mataix-Cols, 2009), an alteration that was shown to be restored in responders to SRI treatment (Atmaca et al., 2006). At the functional level, hyperactivity of the anterior cingulate, orbitofrontal cortex, and caudate (Saxena et al., 2001; Maia et al., 2008), as well as altered functional connectivity between the medial frontal cortex and striatal regions have been reported in OCD (Fitzgerald et al., 2011; Posner et al., 2014).

## Glutamate System Dysfunctions in the Pathogenesis of OCD

Multiple neurotransmitter systems have been implicated in the etiology of OCD. Since clinically efficacious pharmacotherapy targets the serotonin system, impairments in this neurotransmitter were hypothesized to be disease-causing (Insel et al., 1985; March et al., 1989; Barr et al., 1993) and probably explain certain aspects of OCD pathophysiology. However, like other neuropsychiatric entities, OCD is multifactorial and thereby additional hypotheses have been proposed (Pauls et al., 2014). For instance, some SRI-refractory OCD patients can benefit from combined therapy with antipsychotic drugs targeting dopamine D2 receptors (Dold et al., 2013; Dold et al., 2015), consistent with changes in dopaminergic system reported in OCD patients (Denys et al., 2004), including a decrease in dopamine transporter (DAT) and dopamine D2 receptors levels in basal ganglia (Denys et al., 2004; Hesse et al., 2005). Both monoamine hypotheses are supported by reports of OCD-like behaviors in animal models where modifications of serotonin or dopamine neurotransmitter systems were achieved by genetic or pharmacological intervention (Yadin et al., 1991; Szechtman et al., 1998; Chou-Green et al., 2003; Ralph-Williams et al., 2003).

Over the last decades, increasing lines of evidence support a glutamatergic hypothesis in OCD. For instance, multiple trials have reported promising results in OCD using glutamate-targeting drugs. Memantine, ketamine, and rapastinel, all drugs acting on glutamate NMDA receptors (NMDAR) have shown beneficial effects in OCD treatment (Bakhla et al., 2013; Rodriguez et al., 2016; Modarresi et al., 2018). Moreover, Riluzole, a drug that reduces glutamate synaptic levels (by acting at both presynaptically and at glial glutamate transporters) has been shown to improve OCD symptomatology in about 50% of patients when given concomitantly with SRI (Coric et al., 2005; Grant et al., 2007; Pittenger et al., 2015). Other studies have shown that the adjunctive use of N-acetyl cysteine alleviates OCD symptomatology (Lafleur et al., 2006; Lalanne et al., 2014; Ghanizadeh et al., 2017; di Michele et al., 2018). N-acetyl cysteine is a cysteine donor, precursor for glutathione (a potent antioxidant), and also increases glutamate levels by activating the glial glutamate/cysteine antiporter.

Alterations in fluid or imaging biomarkers of glutamatergic neurotransmission have been reported in OCD, including increased glutamate content in cerebrospinal fluid of OCD untreated patients (Chakrabarty et al., 2005) and a positive correlation between compulsive behavior and glutamate concentration in the anterior cingulate cortex (Naaijen et al., 2017). Interestingly, a previous study performed in child and adolescent OCD patients reported decreased, rather than increased glutamate levels in this brain region (Rosenberg et al., 2004). Higher glutamate concentration was also found in the caudate nucleus in child OCD patients that was restored after treatment with paroxetine (Rosenberg et al., 2000). Such alterations in the levels of glutamate in distinct nuclei of the CSTC loop might lead to (or reflect) functional changes that ultimately, lead to abnormal neuronal circuit activity. Consistent with this idea, three recently developed animal models of OCD display glutamatergic alterations in cortico-striatal synapses (Welch et al., 2007; Shmelkov et al., 2010; Delgado-Acevedo et al., 2019).

## Genetics of Glutamatergic System Genes in OCD

Genetic studies also provide support for the glutamatergic hypothesis of OCD, including linkage and association studies. For instance, the 5072T/G variant of *GRIN2B* gene, which encodes for the NR2B subunit of NMDARs, was significantly associated with OCD in a family-based study (Arnold et al., 2004), whereas the rs1019385 polymorphism was associated with reduced glutamate levels in the anterior cingulate in drug-free pediatric OCD patients. Variants in the *GRIK2* gene encoding the kainate receptor subunit 2 have been also reported in OCD (Delorme et al., 2004; Sampaio et al., 2011). Another glutamate-related gene proposed in OCD is *DLGAP3*, which encodes for the postsynaptic scaffolding protein SAPAP3 implicated in the anchoring of glutamate receptors. As discussed below, SAPAP3 knock-out (KO) mice display OCD relevant behaviors (Welch et al., 2007). These findings prompted genetic studies where some *DLGAP3* gene variants were found to be more associated with grooming disorders than with OCD (Bienvenu et al., 2009; Zuchner et al., 2009; Boardman et al., 2011).

Genetic studies have also suggested a role for the *SLC1A1* gene, encoding the neuronal glutamate transporter 3 (EAAT3) in OCD. This gene was originally proposed in two independent genome-wide linkage OCD studies (Hanna et al., 2002; Willour et al., 2004). Subsequent family-based association and case-control studies found *SLC1A1* gene variants that are associated with OCD (Arnold et al., 2006; Dickel et al., 2006; Stewart et al., 2007; Shugart et al., 2009; Wendland et al., 2009; Samuels et al., 2011). In addition, an association between an *SLC1A1* haplotype and the appearance of atypical antipsychotic-induced OCD symptoms has been also reported (Kwon et al., 2009), reinforcing the idea that modifications of *SLC1A1* gene might underlie the generation of compulsive behavior. A recent study found that some *SLC1A1* variants are associated with white matter microstructure modifications in child and adolescent OCD patients (Gasso et al., 2015), suggesting that they might also underlie the anatomical alterations seen in OCD. Nevertheless, a meta-analysis study found just a weak association between the *SLC1A1* variant rs301443 and OCD, while rs12682807 was modestly associated in male subjects (Stewart et al., 2013). The lack of stronger association might be attributed to inadequate sample size, distinct clinical subtypes of OCD and genetic/phenotypic heterogeneity of the subjects (Stewart et al., 2013; Rajendram et al., 2017). Two genome-wide association studies reported no *SLC1A1* variants reaching genome wide significance, potentially due to statistical power limitations given low sample size (Stewart et al., 2013; Mattheisen et al., 2015).

To date, three studies have addressed the impact of *SLC1A1* gene variants on EAAT3 expression/function. Veenstra-VanderWeele and colleagues characterized the rare *SLC1A1* coding variant T164A found in an OCD family, which was shown to have modest effects on both EAAT3 V_max_ and K_m_ parameters, suggesting a decrease in the number of EAAT3 available and its affinity for glutamate, respectively, which might be involved in the disorder (Veenstra-VanderWeele et al., 2012). However, no protein quantification was carried out in this work. In a second study, Bailey and colleagues characterized the EAAT3 loss-of-function variants R445W and I395del found in human dicarboxylic aminoaciduria; both mutants dramatically reduced or abolished glutamate and cysteine transport by EAAT3 and led to almost absent EAAT3 surface expression in a cell line model (Rodenas-Ruano et al., 2012). Although no psychiatric assessment was available for the subjects in this study, authors reported that one of the patients carrying R445W variants exhibited features consistent with a diagnosis of OCD (Bailey et al., 2011). In a third study, Wendland and colleagues described *SLC1A1* variants that affect mRNA expression in human dorsolateral prefrontal cortex tissue and that were associated with OCD in a large case-control study (Wendland et al., 2009). Collectively, these data suggest that gene variants impacting EAAT3 expression might underlie the pathogenesis of OCD. A more definitive answer will hopefully be provided in the near future through adequately-powered, large genome-wide association or sequencing studies that can yield statistically robust insights into the role of *SLC1A1* and other glutamatergic system genes in OCD.

## The Neuronal Glutamate Transporter EAAT3

EAAT3 belongs to the excitatory amino acid transporters family (EAAT1-5) that regulates the extracellular levels of glutamate. Although its expression is approximately 100-fold lower than other EAAT in brain (Holmseth et al., 2012), enriched EAAT3 content is found in CSTC loop, including the cerebral cortex, hippocampus, striatum, and basal ganglia (Rothstein et al., 1994; Furuta et al., 1997; Shashidharan et al., 1997; Kanai and Hediger, 2004; Holmseth et al., 2012), in glutamatergic, GABAergic, and dopaminergic neurons (Coco et al., 1997; Conti et al., 1998; Sidiropoulou et al., 2001; Underhill et al., 2014). EAAT3 is localized peri-synaptically on the postsynaptic spine (Coco et al., 1997; He et al., 2000), and several studies have indicated that its contribution to the overall glutamate uptake is much lesser compared to that of the astrocytic transporters EAAT1 and EAAT2 (Tong and Jahr, 1994; Rothstein et al., 1996; Diamond and Jahr, 1997; Tanaka et al., 1997). Nonetheless, increasing evidence indicate that EAAT3 contributes to buffer glutamate released during synaptic events and prolong the time course of astrocytic clearance in the hippocampus (Scimemi et al., 2009), and to regulate the responses of extra-synaptic glutamate NMDA and AMPA receptors (Scimemi et al., 2009; Jarzylo and Man, 2012; Underhill et al., 2014; Delgado-Acevedo et al., 2019) as well as metabotropic glutamate receptors (Otis et al., 2004; Wadiche and Jahr, 2005). As changes in EAAT3 activity can modify the trafficking of AMPARs and function of NMDARs, it is not surprising that EAAT3 has been shown to be involved in excitatory synaptic plasticity (Cao et al., 2014; Bjorn-Yoshimoto and Underhill, 2016; Delgado-Acevedo et al., 2019). EAAT3 can also control neuronal activity through a negative feedback of excitatory neurotransmission by regulating the synthesis of GABA at inhibitory terminals (Sepkuty et al., 2002; Mathews and Diamond, 2003) and by regulating the expression of D1 dopamine receptors (Bellini et al., 2018). Collectively, the evidence strongly supports the notion that EAAT3 regulates glutamate levels in the synaptic cleft and thus the function of postsynaptic receptors. Therefore, changes in EAAT3 expression and/or function at glutamatergic synapses within the CSTC loop might underlie the neurobiological basis of OCD.

EAAT3 has been also suggested to play a role on the neuronal redox balance as they also transport cysteine, the rate limiting substrate for glutathione synthesis (Himi et al., 2003; Aoyama et al., 2006; Watabe et al., 2007). Indeed, EAAT3 KO mice have reduced glutathione brain levels, which correlates with the brain atrophy and hippocampal neurodegeneration in aged mice and impaired spatial memory reported in these mice (Aoyama et al., 2006; Berman et al., 2011). Increased oxidative stress and a reduced number of dopaminergic neurons were also found in EAAT3 KO mice (Berman et al., 2011). Correspondingly, mice lacking GTRAP3-18, a reticulum protein that interacts with and retains EAAT3 on cellular compartment (Lin et al., 2001) not only showed increased EAAT3 levels at the plasma membrane, but also increased cysteine and glutathione brain content as well as improved performance in spatial memory tasks (Aoyama et al., 2012). In contrast, mice overexpressing EAAT3 have higher glutathione brain levels (Delgado-Acevedo et al., 2019), raising the possibility that alterations in redox balance might be also involved in OCD.

## Impaired Glutamatergic Neurotransmission in OCD Animal Models

Three different genetic models, namely, the Sapap3 KO (Welch et al., 2007) mice, the Slitrk5 KO mice (Shmelkov et al., 2010), and the EAAT3 overexpressing mice (Delgado-Acevedo et al., 2019), have been reported to recapitulate OCD hallmarks including cortico-striatal glutamatergic alterations, particularly changes in NMDAR function, and displaying OCD-like behaviors that could be rescued by chronic administration of SSRI (See **Table 1**). Mice lacking SAPAP3 (a member of SAP90/PSD-95-associated protein family (SAPAPs)) exhibit an aberrantly increased self-grooming leading to skin wounds (Welch et al., 2007). SAPAP3 KO mice also showed increased anxiety-like behavior in the open field, zero-maze, and light-dark emergence tests; both increased anxiety and hyper-grooming behaviors were alleviated by fluoxetine administration for 6 days (Welch et al., 2007). SAPAP3 KO mice also show abnormalities at cortico-striatal synapses, including a decrease in AMPAR-mediated transmission and an increase in NMDAR-mediated transmission. Such alterations were specific for cortico-striatal synapses, as they were not observed in the hippocampus (Welch et al., 2007) neither in thalamo-striatal synapses (Wan et al., 2014) and were rescued by striatal injections of a lentivirus expressing SAPAP3 (Welch et al., 2007). While the reduction in AMPAR-mediated transmission is likely due to AMPAR endocytosis, an increased activity of mGluR5 receptors was also reported and might be involved in the increase of silent synapses (Wan et al., 2011). This increased mGluR5 activity seemed not only to accompany the OCD-like phenotype but could also be a causative agent, since acute mGluR5 activation recapitulated OCD-like behavior in wildtype mice, and mGluR5 antagonism reverted the phenotype on SAPAP3 KO mice (Ade et al., 2016). Additional dysregulation in serotonin and dopamine release and/or metabolism (Wood et al., 2018) as well as deficits in behavioral inhibition have been also reported in SAPAP3 KO mice (Burguiere et al., 2013). While this evidence links SAPAP3 to compulsive behaviors, human *DLGAP3*/SAPAP3 gene variants seem to be more related to disorders belonging to the obsessive-compulsive spectrum rather than directly to OCD, as discussed above (Bienvenu et al., 2009; Zuchner et al., 2009; Boardman et al., 2011).

**Table 1:** Summary of the behavioral alterations relevant to obsessive-compulsive disorder reported in the SAPAP3 KO mouse, Slitrk5 KO mouse, and EAAT3glo/CMKII mouse.

Animal Model	Grooming behavior	Reversion with SSRI	Extinction deficits	Marble burying	Locomotion	Open Field	Elevated Zero-Maze	Elevated Plus Maze	Dark-light emergence	Other behavioral tests
Sapap3 KO (Welch et al., 2007)	Very high (self-injured) starting at 6 months. Increased number of bouts and duration.	Yes, 6 days fluoxetine treatment	Not tested	Not tested	Normal	Less time in center	-Higher latency to open area. -Less time in open area	Not tested	-Higher latency to lit. -Less time in the lit chamber	Impaired behavioral inhibition (tone paired with water drop (Burguiere et al., 2015)
Slitrk5 KO (Shmelkov et al., 2010)	Very high (self-injured) starting at 3 months). Increased duration.	Yes, 21 days fluoxetine treatment	Not tested	Increased	Normal	-Less time in center. Less entries to center	Not tested	Reduced time in open arms	Not tested	–
EAAT3glo/ CMKII (Delgado-Acevedo et al., 2019)	High (no self-injured) tested at 2-3 months. Increased duration.	Yes, 21 days fluoxetine or clomipramine treatment	Greater spontaneous recovery of fear memories	Increased	Normal	-Less time in center. -Less entries to center	Not tested	Not tested	-Higher latency to lit. -Less time in the lit chamber	Unaltered cognitive flexibility in visual discrimination task

Similarly, the deletion of *SlitrK5* gene was found to induce compulsive and anxiety-like behaviors in mice (Shmelkov et al., 2010). The N-terminal region of SlitrK5, a transmembrane protein, is similar to Slit proteins conformed by two leucine-rich domains, whereas the C-terminal is similar to Trk neurotrophin receptor (Aruga and Mikoshiba, 2003). Like SAPAP3 KO mice, increased self-grooming leading to skin lesions and facial hair loss has been reported in SlitrK5 KO mice (Shmelkov et al., 2010). They also display compulsive-like behavior in the marble burying test and increased anxiety-like behavior in the open field and elevated plus maze tests, behavioral impairments that were restored by chronic (18 days) treatment with fluoxetine (Shmelkov et al., 2010). Importantly, SlitrK5 KO mice have selective over-activation of the orbitofrontal cortex and abnormalities in striatal glutamatergic signaling, including a reduction in the level of glutamate receptor subunits GluR1, GluR2, NR2A, and NR2B and a decrease in the amplitude of spikes in response to the cortico-striatal stimulation (Shmelkov et al., 2010). While the exact mechanism by which Slitrk5 deletion impacts glutamatergic signaling remains unclear, Slitrk5 might play an important role in neuronal development as a rare synaptogenesis-impairing mutation in the *SLITRK5* gene has been recently associated with OCD (Song et al., 2017), suggesting that changes in the development and/or maturation of excitatory neurons in the CSTC circuit might underlie OCD pathophysiology. Consistent with this idea, SlitrK5 KO mice display decreased arbor complexity in striatal medium spiny neurons (Shmelkov et al., 2010), which might explain the decreased number of postsynaptic glutamate receptors and glutamatergic function, two hallmarks of OCD disorders.

Mouse models with altered EAAT3 expression have been useful to study OCD neurobiology. The first EAAT3 KO mouse model was reported in 1997 and indicated reduced spontaneous locomotor activity, but no other neurological dysfunctions nor OCD relevant behaviors (Peghini et al., 1997). Later, it was found that EAAT3 KO mice also display an age-dependent decrease of dopaminergic neurons number in the substantia nigra, which might explain the impaired locomotor activity (Berman et al., 2011). Similarly, changes in the volume of the hippocampal CA1 region, associated with an age-progressive decline in swim velocity and in spatial memory performance were also reported (Aoyama et al., 2006). Moreover, neurons from EAAT3 KO mice have been found to be more sensitive to oxidative stress (Aoyama et al., 2006), ischemia (Jang et al., 2012; Choi et al., 2014), and traumatic brain injury (Choi et al., 2016). Since some of the alterations in EAAT3 KO mice can be rescued by the treatment with N-acetyl cysteine (Berman et al., 2011), it is plausible to suggest that EAAT3 has a role in neuroprotection due to its cysteine donor function. In this context, increased oxidative status in OCD patients as well as a positive correlation between the severity of OCD symptoms and the level of lipid peroxidation have been reported (Chakraborty et al., 2009). Increased glutathione peroxidase levels in the plasma serum of treatment-naïve children with OCD (Simsek et al., 2016) and lower levels of glutathione in the posterior cingulate cortex have been also reported (Brennan et al., 2016). While animal and human studies indicate that SRIs can restore neuron oxidative status observed in mood disorders, to the best of our knowledge, only one study has suggested that fluoxetine treatment restores the oxidative status in OCD patients (Chakraborty et al., 2009).

After two decades of research since the first publication of an EAAT3 KO mouse model (Peghini et al., 1997), only one study has reported some behaviors reminiscent of compulsive-like behavior in EAAT3 KO mice (Bellini et al., 2018). A second EAAT3 KO mouse model was recently developed by a different research group and, in agreement with the majority of the studies, they found unaltered anxiety or compulsive-like behaviors in this model (Zike et al., 2017). Indeed, a decrease in grooming behavior and amphetamine-induced locomotor activity, as well as in D1 dopamine receptor agonist-induced stereotypy (an effect correlated with reduced dopamine release in the striatum) was reported in this second EAAT3 KO model (Quinlan et al., 1999). While genetic background (C57BL/6 vs CD1 strain) and/or the age of the animals might explain some of the differences observed in the repetitive behavior in EAAT3 KO mouse, both studies highlight the impact of EAAT3 ablation on dopamine neurotransmission (Zike et al., 2017; Bellini et al., 2018). Altogether, these data suggest that OCD susceptibility might not be related to a reduced (or absent) EAAT3 expression/function. Consistent with this idea, one of the *SLC1A1* gene variants highly replicated in OCD genetic studies is associated with increased rather than decreased EAAT3 mRNA levels in human brain tissue (Wendland et al., 2009). To explore this EAAT3 gain-of-function hypothesis, a conditional transgenic mouse line, in which EAAT3 was overexpressed under CamKIIα driver (EAAT3glo/CMKII) was recently reported (Delgado-Acevedo et al., 2019). This model exhibits increased anxiety and compulsive-like behaviors that were restored by chronic, but not acute, fluoxetine, or clomipramine treatment. Moreover, EAAT3glo/CMKII mice displayed alterations in the glutamatergic system in the striatum, including changes in NMDARs subunit composition (increased NR2B/NR2A ratio), and impaired NMDA-dependent synaptic plasticity (Delgado-Acevedo et al., 2019). Interestingly, EAAT3glo/CMKII mice also displayed higher spontaneous recovery of fear memory, suggesting impairment in long-term extinction, which has also been described in OCD patients (Milad et al., 2013) and, to the best of our knowledge, for the first time in an OCD mouse model (Delgado-Acevedo et al., 2019). In view of their high density in extinction-related brain areas, the NMDAR has been suggested to be a candidate for modulating extinction learning (for review see (Myers and Davis, 2002; Davis, 2011). However, whether changes in NMDAR subunits composition observed in the striatum of EAAT3glo/CMKII mice (Delgado-Acevedo et al., 2019) also occur in extinction-related areas remain to be determined.

## Conclusions

Converging evidence from genetic, neuroanatomical, pharmacological, and preclinical studies in both humans and animal models support that glutamate dysregulations may contribute to the pathophysiology of OCD. As discussed above, the findings from genetic mouse models that are relevant of OCD not only highlight glutamatergic alterations, and particularly altered NMDAR function in the OCD etiology, but also establish EAAT3 overexpression rather than an EAAT3 reduction as an attractive model to further explore in this and other related disorders. While the exact mechanism by which altered EAAT3 levels impact NMDARs subunit composition remains unknown, the fact that they can change in the order of minutes to hours in response to neuronal activity (Barria and Malinow, 2002; Bellone and Nicoll, 2007; Matta et al., 2011), as well as in a long-lasting manner by early life experience (Quinlan et al., 1999; Rodenas-Ruano et al., 2012) strongly suggests that EAAT3 not only limits NMDARs function by rapidly binding synaptically released glutamate (Scimemi et al., 2009), but also might lead to a cascade of events that, ultimately, can modify the relative levels of NR2B to NR2A subunits composition of NMDARs in a long-lasting manner during brain development to impair function. Since redox balance also alters NMDAR function (Choi and Lipton, 2000), the EAAT3-dependent cysteine uptake might also impact glutamatergic neurotransmission by modifying the neuronal oxidative status. Further studies are warranted to better dissect the interplay between of NMDAR and redox balance in the etiology of OCD.

## Author Contributions

PM conceived the work. AE, JW, AC, PM drafted the work, critically revised the work and provided important intellectual content. AE, JW, AC, PM provided approval for publication of the content and agreed to be accountable for all aspects of the work.

## Funding

This work was supported by grants Fondecyt N° 1190833, Fondecyt N° 3190843, and ICM-MINECOM N° P09-022-F CINV.

## Conflict of Interest

The authors declare that the research was conducted in the absence of any commercial or financial relationships that could be construed as a potential conflict of interest.

The handling editor declared a shared affiliation, though no other collaboration, with the authors AE, JW, AC, PM at time of review.
